# Research Note: Detection of *Campylobacter* spp. in chicken meat using culture methods and quantitative PCR with propidium monoazide

**DOI:** 10.1016/j.psj.2023.102883

**Published:** 2023-06-18

**Authors:** Ayaka Okada, Mizuki Tsuchida, Kazuha Aoyagi, Ayaka Yoshino, Md. Matiur Rahman, Yasuo Inoshima

**Affiliations:** ⁎ Laboratory of Food and Environmental Hygiene, Cooperative Department of Veterinary Medicine, Faculty of Applied Biological Sciences, Gifu University, Gifu 501-1193, Japan; † Education and Research Center for Food Animal Health, Gifu University (GeFAH), Gifu 501-1193, Japan; ‡ Department of Medicine, Faculty of Veterinary, Animal and Biomedical Sciences, Sylhet Agricultural University, Sylhet 3100, Bangladesh; § Joint Graduate School of Veterinary Sciences, Gifu University, Gifu 501-1193, Japan

**Keywords:** Campylobacter, culture method, propidium monoazide, quantitative PCR, viable but nonculturable

## Abstract

Globally, *Campylobacter* spp. are prominent causative agents of food-borne gastroenteritis. These pathogens are commonly detected using conventional culture methods; however, culture methods are unable to detect viable but nonculturable (**VBNC**) bacteria. Currently, the detection rate of *Campylobacter* spp. on chicken meat does not correlate with the seasonal peak of human campylobacteriosis. We hypothesized that this may be due to the presence of undetectable VBNC *Campylobacter* spp. Therefore, we previously established a quantitative PCR assay using propidium monoazide (**PMA-qPCR**), which can detect viable *Campylobacter* cells. In this study, PMA-qPCR was conducted to detect viable *Campylobacter* spp. in chicken meat, and the detection rates of PMA-qPCR and the culture method throughout all 4 seasons were compared. A total of 105 chicken meat samples (whole legs, breast fillets, and livers) were screened for the presence of *Campylobacter* spp. using both PMA-qPCR and the conventional culture method. The detection rates of the 2 methods did not differ significantly; however, the positive and negative samples were not always consistent. Detection rates in March were significantly lower compared to months with the highest detection rates. These results suggest that, to increase the detection rate of *Campylobacter* spp., the 2 methods should be used in parallel. In this study, PMA-qPCR could not detect VBNC *Campylobacter* spp. effectively in *C. jejuni*-spiked chicken meat. Further studies using improved viability-qPCR should be performed to describe the impact of the VBNC state of *Campylobacter* spp. on the detection of this bacterium in chicken meat.

## INTRODUCTION

*Campylobacter* spp. are the leading cause of foodborne bacterial gastroenteritis worldwide and is mostly associated with contaminated chicken meat (Skirrow, 1977). According to statistics from the Japanese Ministry of Health, Labour, and Welfare, *Campylobacter jejuni* and *Campylobacter coli* have been the predominant cause of bacterial food poisoning in Japan since 2003 (https://www.mhlw.go.jp/stf/seisakunitsuite/bunya/kenkou_iryou/shokuhin/syokuchu/04.html [accessed on March 14, 2023]).

Globally, conventional culture methods are commonly used to detect *Campylobacter* spp. in poultry. However, exposure to various environmental stressors, such as low temperature, nutrient starvation, and unsuitable aerobic conditions can cause *Campylobacter* spp. to enter a viable but nonculturable (**VBNC**) state (Yagi et al., 2022). PCR or quantitative PCR (**qPCR**) are useful alternatives to conventional culture methods for the detection of both culturable and VBNC organisms. However, both PCR and qPCR can amplify bacterial DNA originating from nonviable cells that will not cause food poisoning. To solve this problem, propidium monoazide (**PMA**), which only enters dead bacteria through the damaged cell wall/cell membrane, binds to the DNA, and prevents DNA amplification via PCR, has been combined with PCR or qPCR to detect only viable bacteria (Okada et al., 2022).

The detection rate of *Campylobacter* spp. in poultry varies by season, increasing in warmer seasons and dropping during the winter (Meldrum et al., 2005). However, the number of human campylobacteriosis case in Japan reaches a maximum in June, preceding the increase in *Campylobacter*-contaminated chicken meat samples observed from June to November (Ishihara et al., 2012). Therefore, the variation in the amount of *Campylobacter* spp. detected in chicken meat is suggested to not be the primary reason for the seasonality of human campylobacteriosis (Meldrum et al., 2005, Williams et al., 2015). We hypothesized that the low detection rate in winter might be due to the presence of VBNC *Campylobacter* spp. that remain undetected by conventional culturing methods.

Previously, we optimized a protocol for PMA treatment followed by qPCR (**PMA-qPCR**) to distinguish between dead and living cells (Okada et al., 2022). Our results suggested that the optimized PMA-qPCR was able to detect VBNC *Campylobacter* spp. in chicken meat; however, the previous experiment was only conducted for one month. Therefore, in this study, we aimed to compare PMA-qPCR with conventional culture methods and to examine the ability of PMA-qPCR to detect the VBNC state of *Campylobacter* spp. in chicken meat throughout the year.

## MATERIALS AND METHODS

### Chicken Meat

A total of 105 chicken meat packs were purchased from 4 local supermarkets in the Gifu Prefecture, Japan, from September 2021 to June 2022. The following samples were collected: whole legs (*n* = 36), breast fillets (*n* = 36), and livers (*n* = 33). Samples were transported to the laboratory in isothermal boxes and bacterial isolation was performed within 1 h after purchase.

### PMA-qPCR

To perform PMA-qPCR, 25 g of each chicken sample was rinsed with 10 mL of sterilized PBS. The rinsed solution was centrifuged at 800 × *g* for 10 min at 4°C to eliminate larger debris. The supernatant was then centrifuged at 8,000 × *g* for 10 min at 4°C to pellet bacteria. Cells were subjected to a 2-round PMA treatment, as described previously (Okada et al., 2022). Briefly, each pellet was resuspended in 160 µL of PBS, followed by the addition of 40 µL of PMA Enhancer for gram-negative Bacteria (5X solution; Biotium Inc., Fremont, CA; final concentration: 1X) and 0.5 µL of PMAxx solution (20 mM in H_2_O; Biotium Inc.; final concentration: 25 μM). Each sample was thoroughly mixed and incubated in the dark at 37°C for 10 min, followed by light exposure for 15 min using an LED Crosslinker 12 (Takara, Kusatsu, Japan) to activate the PMAxx. Treated cells were centrifuged at 8,000 × *g* for 10 min at 4°C and the supernatant was removed. A second round of PMA treatment was performed as described above. Cells were collected using centrifugation at 8,000 × *g* for 10 min at 4°C, and DNA was extracted from each pellet using a DNeasy Blood & Tissue Kit (Qiagen, Hilden, Germany) according to the manufacturer's instructions. Extracted DNA was subjected to qPCR using a StepOnePlus thermal cycler (Applied Biosystems, Foster City, CA). To detect *Campylobacter* spp., specific primers, campF2 and campR2, and a probe, camp2probe, were used (Lund et al., 2004). The PCR mixture was prepared to a final volume of 20 µL, which contained 2 µL of the extracted DNA, using the GoTaq Probe qPCR Master Mix (Promega, Madison, WI), according to the manufacturer's instructions. Thermal cycling was performed under the following conditions: 2 min at 95ºC, followed by 40 cycles of 95ºC for 3 s and 60ºC for 30 s. Fluorescence was measured at the end of each cycle.

### Culture-Based Enumeration

The standard method for detection of *Campylobacter* spp. in Japan, following the NIHSJ-02 guideline, was used (Momose et al., 2013). Approximately 25 g of each chicken sample was added to 100 mL of Preston selective broth comprised of nutrient broth No. 2 (Oxoid, Hampshire, UK), 5% of sterile lysed horse blood, *Campylobacter* growth supplement (Oxoid), and modified Preston *Campylobacter* selective supplement (Oxoid). Each sample was mixed for 1 min using a stomacher and then incubated at 42°C for 24 h under microaerobic conditions. Enriched cultures were streaked on modified charcoal-cefoperazone-deoxycholate agar (**mCCDA**) and Skirrow agar comprised of blood agar base No. 2 (Oxoid), 7% of sterile lysed horse blood, Skirrow *Campylobacter* selective supplement (Oxoid), and *Campylobacter* growth supplement. Streaked cultures were incubated at 42°C for 48 h under microaerobic conditions. A colony resembling *Campylobacter* spp. was selected from each of the samples and identified using colony PCR with C412F and C1228R primers (Linton et al., 1996). Amplicons were purified using ethanol precipitation and sequenced using the C412F and C1228R primers in an ABI 310 Genetic Analyzer with the Big Dye Terminator v3.1 Cycle Sequencing Kit (Applied Biosystems). Sequences were subjected to BLAST verification using the NCBI database.

### *Campylobacter Jejuni*-Spiked Chicken Meat

*Campylobacter jejuni* NCTC11168 strain was purchased from the American Type Culture Collection (ATCC 700819). To generate culturable *C. jejuni*, cells from the -80°C stock were cultured on mCCDA at 37°C for 2 d under microaerobic conditions. Colonies were suspended in 10 mL of Brucella broth and cultured under microaerobic conditions at 37°C for 36 h at 130 rpm using an incubator shaker (Innova 4300, New Brunswick Scientific, Enfield, CT). *C. jejuni* cells were resuspended in PBS and diluted to an optical density at 600 nm (OD_600_) of 1.0 using a spectrophotometer (GeneQuant 100, GE Healthcare, Chicago, IL), which corresponded to approximately 1×10^8^ CFU/mL. To generate VBNC *C. jejuni*, culturable cells were resuspended in Muller-Hinton (MH) broth (275730, BD Biosciences, Billerica, MA), adjusted to 1 × 10^8^ CFU /mL, and cultured at 4°C for 30 d under aerobic conditions as previously described (Yagi et al., 2022). Cell viability was examined using a LIVE/DEAD BacLight Bacterial Viability Kit (Invitrogen, Carlsbad, CA).

Liquid cultures from culturable and VBNC cells were adjusted to 1 × 10^6^ CFU/mL via serial dilution of the stock solution (1 × 10^8^ CFU/mL). To generate the *C. jejuni*-spiked chicken meat, 100 µL of 1 × 10^6^ CFU/mL bacterial suspension was applied to 25 g of chicken meats (whole legs). The meat was confirmed a priori to be *Campylobacter* spp. free using both culture method and PMA-qPCR. *C. jejuni*-spiked chicken meat was put in the refrigerator overnight to simulate *Campylobacter* spp. contaminated chicken meat. Culturable and VBNC *C. jejuni* were detected via qPCR with and without PMA treatment.

### Statistical Analysis

Statistical analysis of results was performed using McNemar's and chi-square tests (Bonferroni correction). Results with *P*-values < 0.05 were considered significant.

## RESULTS AND DISCUSSION

We compared PMA-qPCR with culture methods for the detection of *Campylobacter* spp. in chicken meat. The prevalence of *Campylobacter* spp. in commercial chicken meat as detected by both methods is summarized in Table 1. No significant difference was found between the detection rate of PMA-qPCR and that of the culture method for whole legs, breast fillets, and livers. The number of positive and negative results determined using both methods is summarized in a 2 × 2 matrix (Table 2). The results obtained using the 2 methods were not consistent.

**Table 1:** Detection of *Campylobacter* spp. in chicken meat using a quantitative PCR assay with propidium monoazide (PMA-qPCR) or conventional culture methods.

Chicken part	Detection method	March	June	September^1^	December	Total
**Whole leg**	PMA-qPCR	2/9 (22%)	3/9 (33%)	9/9 (100%)	5/9 (56%)	19/36 (53%)
	Culture	1/9 (11%)	5/9 (56%)	8/9 (89%)	8/9 (89%)	22/36 (61%)
**Breast fillet**	PMA-qPCR	1/9 (11%)	1/9 (11%)	4/9 (44%)	3/9 (33%)	9/36 (25%)
	Culture	2/9 (22%)	6/9 (67%)	3/9 (33%)	5/9 (56%)	16/36 (44%)
**Liver**	PMA-qPCR	4/8 (50%)	5/8 (63%)	6/8 (75%)	3/9 (33%)	18/33 (55%)
	Culture	1/8 (16%)	8/8 (100%)	3/8 (38%)	2/9 (22%)	14/33 (42%)
**Total**	PMA-qPCR	7/26 a (27%)	9/26 a,b (35%)	19/26 b (73%)	11/27 a,b (41%)	46/105 (44%)
	Culture	4/26 a (15%)	19/26 b (73%)	14/26 a,b (54%)	15/27 b (56%)	52/105 (50%)

Results are expressed as number of *Campylobacter*-positive samples per number of samples analyzed (%). The differences between the detection rates of PMA-qPCR and culture were evaluated using the McNemar's test. Seasonality was evaluated through Chi-square test (Holm-Bonferroni correction).

a,b values within rows with different superscripts differed significantly (*P* < 0.05).

1 The results of September were data from the previous report (Okada et al., 2022).

**Table 2:** Comparison between quantitative PCR assay with propidium monoazide (PMA-qPCR) and conventional culture methods for detection of *Campylobacter* spp. in chicken samples.

		PMA-qPCR		
		Positive	Negative	Total
Culture	Positive	33	19	52
	Negative	13	40	53
	Total	46	59	105

In samples that tested positive using PMA-qPCR, but negative using the culture method, we predicted that *Campylobacter* spp. were in a VBNC state. To test whether PMA-qPCR could effectively detect VBNC *Campylobacter* spp., we performed experiments using culturable and VBNC *C. jejuni*-spiked chicken meat. The viability of *C. jejuni* was confirmed to be more than 80% in both culturable and VBNC cells. VBNC cells were confirmed not to form colonies on mCCDA. Culturable and VBNC *C. jejuni* cells were effectively detect via qPCR without PMA treatment (Ct values ranging from 21 to 23). Conversely, using PMA-qPCR, VBNC state of *C. jejuni* was not effectively detect, presenting Ct values above 35 or ‘‘not detected’’, whereas culturable *C. jejuni* was effectively detected (Ct values ranging from 23 to 24). These results indicated that PMA-qPCR not always detected VBNC cells. Therefore, there might be 2 reasons for *Campylobacter* spp. not being detected using the culture method but detected via PMA-qPCR. First, *Campylobacter* spp. were in VBNC state. Second, the growth of *Campylobacter* spp. in culture media was affected by some substances present in chicken meat. To detect VBNC state of *C. jejuni* effectively, an improved viability-qPCR should be established.

Samples that were positive using both methods could have contained bacteria in both a culturable and VBNC state. In PMA-qPCR-negative but culture-positive samples, a relatively small number of *Campylobacter* spp. were detected using the culture method, which could be attributed to the use of enriched media. As the number of *Campylobacter* spp. in the chicken meat was not counted, quantitative tests should be performed to confirm this hypothesis. To increase the detection rate of *Campylobacter* spp., the 2 methods may be used in parallel (Table 2). However, as *C. jejuni* doses of more than 800 CFU is required for human infection (Black et al., 1988), detecting a small number of bacteria is not feasible, which can only be detected using enrichment culture methods.

Seasonal changes in the detection of *Campylobacter* spp. were evaluated. The percentage of *Campylobacter*-positive chicken meat samples in each month is listed in Table 1. The highest detection rate using PMA-qPCR was observed in September (73%), whereas the culture method identified the most positive samples in June (73%). The lowest detection rate was observed in March using both PMA-qPCR and the culture method (27% and 15%, respectively). The detection rate in March was significantly lower than in September (using PMA-qPCR), and in June and December (using culture methods) (Table 1). An increase in the incidence of *Campylobacter*-positive poultry samples in warmer seasons has been reported, both in other countries ( Meldrum et al., 2005) and in Japan (Ishihara et al., 2012). Our data were consistent with previous studies. We hypothesized that a lower detection rate during colder months could be due to the inability of conventional culture methods to detect *Campylobacter* spp. that have entered the VBNC state induced by low temperatures (Yagi et al., 2022). However, because PMA-qPCR in this study could not detect VBNC *Campylobacter* spp. effectively, further experiments are needed to optimize it.

In conclusion, this study demonstrated that there is no difference between the detections rates of *Campylobacter* spp. in chicken meat using PMA-qPCR and the culture method. Furthermore, our results indicate that the lower detection rate of *Campylobacter* spp. occurs in winter season using culture method and PMA-qPCR. However, PMA-qPCR could not detect VBNC *Campylobacter* spp. effectively. Further studies using improved viability-qPCR should be performed to describe the impact of the VBNC state of *Campylobacter* spp. on the detection of this bacterium in chicken meat.
